# Associations of Sex, Race, and Apolipoprotein E Alleles With Multiple Domains of Cognition Among Older Adults

**DOI:** 10.1001/jamaneurol.2023.2169

**Published:** 2023-07-17

**Authors:** Skylar Walters, Alex G. Contreras, Jaclyn M. Eissman, Shubhabrata Mukherjee, Michael L. Lee, Seo-Eun Choi, Phoebe Scollard, Emily H. Trittschuh, Jesse B. Mez, William S. Bush, Brian W. Kunkle, Adam C. Naj, Amalia Peterson, Katherine A. Gifford, Michael L. Cuccaro, Carlos Cruchaga, Margaret A. Pericak-Vance, Lindsay A. Farrer, Li-San Wang, Jonathan L. Haines, Angela L. Jefferson, Walter A. Kukull, C. Dirk Keene, Andrew J. Saykin, Paul M. Thompson, Eden R. Martin, David A. Bennett, Lisa L. Barnes, Julie A. Schneider, Paul K. Crane, Timothy J. Hohman, Logan Dumitrescu

**Affiliations:** 1 Vanderbilt Memory & Alzheimer’s Center, Vanderbilt University Medical Center, Nashville, Tennessee; 2 Vanderbilt Genetics Institute, Vanderbilt University Medical Center, Nashville, Tennessee; 3 Department of Medicine, University of Washington, Seattle; 4 Department of Psychiatry and Behavioral Sciences, University of Washington School of Medicine, Seattle; 5 Geriatric Research Education and Clinical Center (GRECC), VA Puget Sound Health Care System, Seattle, Washington; 6 Department of Neurology, Boston University Chobanian & Avedisian School of Medicine, Boston, Massachusetts; 7 Cleveland Institute for Computational Biology, Department of Population and Quantitative Health Sciences, Case Western Reserve University, Cleveland, Ohio; 8 John P. Hussman Institute for Human Genomics, University of Miami Miller School of Medicine, Miami, Florida; 9 Department of Psychiatry, Washington University School of Medicine, St Louis, Missouri; 10 NeuroGenomics and Informatics Center, Washington University School of Medicine, St Louis, Missouri; 11 Department of Biostatistics, Epidemiology, and Informatics, University of Pennsylvania Perelman School of Medicine, Philadelphia; 12 Penn Neurodegeneration Genomics Center, Department of Pathology and Laboratory Medicine, University of Pennsylvania Perelman School of Medicine, Philadelphia; 13 Department of Biostatistics, Boston University School of Public Health, Boston, Massachusetts; 14 Department of Medicine (Biomedical Genetics), Boston University Chobanian & Avedisian School of Medicine, Boston, Massachusetts; 15 Department of Epidemiology, School of Public Health, University of Washington, Seattle; 16 Department of Laboratory Medicine and Pathology, University of Washington, Seattle; 17 Department of Radiology and Imaging Services, Indiana University School of Medicine, Indianapolis; 18 Department of Medical and Molecular Genetics, Indiana University School of Medicine, Indianapolis; 19 Keck School of Medicine, University of Southern California, Los Angeles; 20 Rush Alzheimer’s Disease Center, Rush University Medical Center, Chicago, Illinois

## Abstract

**Importance:**

Sex differences are established in associations between apolipoprotein E (*APOE*) ε4 and cognitive impairment in Alzheimer disease (AD). However, it is unclear whether sex-specific cognitive consequences of *APOE* are consistent across races and extend to the *APOE* ε2 allele.

**Objective:**

To investigate whether sex and race modify *APOE* ε4 and ε2 associations with cognition.

**Design, Setting, and Participants:**

This genetic association study included longitudinal cognitive data from 4 AD and cognitive aging cohorts. Participants were older than 60 years and self-identified as non-Hispanic White or non-Hispanic Black (hereafter, White and Black). Data were previously collected across multiple US locations from 1994 to 2018. Secondary analyses began December 2021 and ended September 2022.

**Main Outcomes and Measures:**

Harmonized composite scores for memory, executive function, and language were generated using psychometric approaches. Linear regression assessed interactions between *APOE *ε4 or *APOE* ε2 and sex on baseline cognitive scores, while linear mixed-effect models assessed interactions on cognitive trajectories. The intersectional effect of race was modeled using an *APOE* × sex × race interaction term, assessing whether *APOE* × sex interactions differed by race. Models were adjusted for age at baseline and corrected for multiple comparisons.

**Results:**

Of 32 427 participants who met inclusion criteria, there were 19 007 females (59%), 4453 Black individuals (14%), and 27 974 White individuals (86%); the mean (SD) age at baseline was 74 years (7.9). At baseline, 6048 individuals (19%) had AD, 4398 (14%) were *APOE *ε2 carriers, and 12 538 (38%) were *APOE *ε4 carriers. Participants missing *APOE* status were excluded (n = 9266). For *APOE *ε4, a robust sex interaction was observed on baseline memory (β = −0.071, SE = 0.014; *P* = 9.6 × 10^−7^), whereby the *APOE *ε4 negative effect was stronger in females compared with males and did not significantly differ among races. Contrastingly, despite the large sample size, no *APOE *ε2 × sex interactions on cognition were observed among all participants. When testing for intersectional effects of sex, *APOE *ε2, and race, an interaction was revealed on baseline executive function among individuals who were cognitively unimpaired (β = −0.165, SE = 0.066; *P* = .01), whereby the *APOE *ε2 protective effect was female-specific among White individuals but male-specific among Black individuals.

**Conclusions and Relevance:**

In this study, while race did not modify sex differences in *APOE *ε4, the *APOE* ε2 protective effect could vary by race and sex. Although female sex enhanced ε4-associated risk, there was no comparable sex difference in ε2, suggesting biological pathways underlying ε4-associated risk are distinct from ε2 and likely intersect with age-related changes in sex biology.

## Key Points

**Question:**

Does sex modify associations of *APOE* ε2 and ε4 with cognition, and do these sex effects differ in non-Hispanic Black and non-Hispanic White individuals?

**Findings:**

This genetic association study found that among 32 427 older adults from different racial groups, females showed stronger negative *APOE* ε4 effects on memory, which did not statistically differ by race. Contrastingly, no sex effects were observed with *APOE* ε2, aside from an intersectional effect of race and sex, whereby the *APOE* ε2 protective effect on executive function was limited to non-Hispanic White females and non-Hispanic Black males.

**Meaning:**

Sex differences in *APOE* associations with cognition are largely limited to ε4 and do not differ by race.

## Teaser

This genetic association study analyzes data from 4 Alzheimer disease and cognitive aging cohorts to investigate whether sex and race modify associations of *APOE* ε4 and ε2 with cognition.

## Introduction

Apolipoprotein E (*APOE*) is the strongest genetic risk factor for late-onset Alzheimer disease (AD) with the ε2 allele reducing risk and ε4 increasing risk.^1,2,3,4,5,6^ Interestingly, associations between *APOE *ε4 and AD differ by sex, whereby associations are stronger among females compared with males.^7,8,9,10,11,12,13,14^ Likewise, *APOE *ε4 is associated with greater memory decline in females with both normal cognition and mild cognitive impairment (MCI),^12,13^ and although *APOE *ε2 effects on cognition have not been as thoroughly examined, 1 recent study showed female ε2 carriers had enhanced neuroprotection on memory performance compared with male ε2 carriers.^14^ Regarding racial differences, several studies have demonstrated that although ε4 frequency is higher in Black participants, associations between *APOE *ε4 and AD risk are weaker in non-Hispanic Black compared with non-Hispanic White individuals (hereafter, Black and White).^15,16,17^

Nevertheless, studies of *APOE* effects on cognition are inconsistent. Some studies suggest strong associations between *APOE *ε4 and rate of cognitive decline in Hispanic^16,18,19^ and African American populations,^20^ whereas other studies report no significant associations between *APOE *ε4 and cognition in these groups.^21,22,23^ As a result, relations among *APOE* (especially *APOE *ε2) and cognition in racially and ethnically diverse groups remain unclear. The sex- and race-specific effects have important implications for ongoing AD clinical trials, where *APOE* has been included as a screening criterion in some trials, and both outcomes and adverse events (such as amyloid-related imaging abnormalities) have been reported to differ by *APOE* genotype in previous trials.^24,25,26,27,28^

Progressive cognitive decline in AD is characterized by memory loss and impairments in executive functioning and language. These correlated domains have some unique and shared genetic architecture, which is important to uncover. Prior studies have assessed *APOE* effects on cognitive domains,^29,30,31,32,33,34^ with several demonstrating strong *APOE* effects on memory.^29,32,33^ In contrast, studies of *APOE* effects on executive function and language are inconsistent with some studies suggesting *APOE *ε4 carriers perform better on executive function tests,^32,33^ whereas others report *APOE *ε4 carriers have more rapid executive function decline.^34^ Regarding language function, 1 study suggested *APOE *ε4 carriers have greater rates of decline in language performance.^34^ Yet neither *APOE *ε2 effects nor intersectional effects of race, sex, and *APOE* on cognition have been thoroughly examined.

The goal of this study is to extend previous work by providing a comprehensive picture of the modifying effect of sex on *APOE *ε2 and *APOE *ε4 associations with domain-specific cognitive trajectories over the course of aging and AD. In 4 well-characterized cohorts of Black (n = 4453) and White (n = 27 974) older adults, we leverage 3 harmonized cognitive domains (memory, executive function, and language) using robust longitudinal data analysis to characterize the intersectional effects of race, sex, and *APOE* on cognitive performance along the spectrum of clinical disease. These results provide the most comprehensive picture to date of sex and racial differences in allele-specific associations of *APOE* with late-life cognitive performance.

## Methods

### Participants

Data were obtained from 4 cohort studies of cognitive aging and AD: the Alzheimer’s Disease Neuroimaging Initiative (ADNI)^35^; the 3 harmonized cohorts of the Religious Orders Study, Rush Memory and Aging Project, and Minority Aging Research Study (ROS/MAP/MARS)^36,37^; the National Alzheimer Coordinating Center (NACC)^38,39,40,41,42^; and the Adult Changes in Thought (ACT) study.^43^ The ADNI and NACC studies enrolled individuals with normal cognition, MCI, or AD, while ROS/MAP/MARS and ACT enrolled participants without known dementia. Participants older than 60 years at baseline were included in this analysis. Sex and race were self-reported. Written informed consent was obtained from all participants in each study, and research was carried out with protocols approved by each site’s institutional review board. These secondary analyses were approved by the Vanderbilt University Medical Center institutional review board. The study followed the Strengthening the Reporting of Genetic Association Studies (STREGA) reporting guidelines.

### Harmonization of Cognitive Measurements

Harmonization of cognitive data was previously described.^44^ We obtained granular data along with detailed documentation on each item in the neuropsychological protocol from each study, such as test versions, stimuli administered, and response coding. Then, a panel of neuropsychologists and neurologists (E.H.T., J.B.M., A.J.S., P.K.C.) assigned test items to a single primary domain: memory, executive function, language, visuospatial ability, or none of these. Differing versions and administration methods were noted at each time point. Next, quality control steps included parsing out missingness codes, recoding, and reverse coding data as needed. We selected the most recent visit for each participant as the reference for co-calibration. These choices enabled us to optimize the spread of cognitive abilities in the data set, which is desirable for ensuring parameters are valid over the entire range of ability levels^45,46^ and still including only a single observation per study participant.

We treated each cognitive item as an ordinal indicator of a domain. Common test items administered and scored the same way across ACT, ADNI, and ROS/MAP/MARS studies served as anchor items to facilitate co-calibration. We used confirmatory factor analysis models with robust maximum likelihood estimation that is robust to missing data in Mplus version 7.4^47^ to co-calibrate separate scores for memory, executive function, and language. We extracted item parameters (loadings and thresholds) for all items, which were used to derive scores in each legacy study separately. These resulted in scores on the same metric.

### *APOE* Genotyping

*APOE* haplotypes for NACC and ACT were determined from the single-nucleotide variants rs7412 and rs429358^48^ and from pyrosequencing of *APOE* codons 112 and 158 for ADNI and ROS/MAP/MARS.^49^

### Statistical Analyses

Statistical analyses were performed using RStudio version 4.1.2. *APOE *ε2 and *APOE *ε4 were modeled separately using dominant models for *APOE *ε2 (because of the small ε2 homozygote sample size, especially when stratified by sex/race) and additive models for *APOE *ε4. We performed analyses in all participants and restricted to participants who were cognitively unimpaired at their first cognitive visit. We used linear regression for each domain to assess baseline cross-sectional *APOE* effects. We used linear mixed-effects regression restricted to those with at least 2 cognitive visits, with time (years from baseline) and the intercept as fixed and random effects. All models covaried for mean-centered age at first cognitive visit. Longitudinal models covaried for age at first visit × time. To test the modifying effect of sex, we included 2-way interactions (ie, sex × *APOE*) in cross-sectional models, and 3-way interactions (ie, sex × *APOE* × time) in longitudinal models. Sex-interaction models were also run stratified by race. The intersectional effects of both race and sex were assessed using a sex × *APOE* × race interaction in cross-sectional analysis and sex × *APOE* × race × time interaction in longitudinal models. Significant results were rerun in each cohort separately to assess consistency (eFigures 1 and 2 in Supplement 1). Corrections for multiple comparisons were performed using the Benjamini-Hochberg false discovery rate (FDR) procedure, accounting for all main effects and interactions modeled. Uncorrected *P* values are presented in the text, and *P* values that passed FDR correction are indicated in the tables.

Sensitivity analyses excluded ε2/ε4 individuals (n = 866), modeled *APOE *ε2 additively, stratified by age (≥75 or <75 years), covaried for education, or covaried for genetic ancestry via the first 5 principal components.

## Results

### Participant Characteristics

Participant characteristics are presented in Table 1 and by cohort in eTable 1 in Supplement 2. Participants missing *APOE* status were excluded (n = 9266). Of 32 427 participants who met inclusion criteria, there were 19 007 females (59%), 13 420 (41%) males, 4453 Black individuals (14%), and 27 974 White individuals (86%); the mean (SD) age at baseline was 74 years (7.9). At baseline, 6048 individuals (19%) had AD, 4398 (14%) were *APOE *ε2 carriers, and 12 538 (38%) were *APOE *ε4 carriers. Higher proportions of females were cognitively unimpaired at study entry, and *APOE* allele frequency differed by race.

**Table 1.** Participant Characteristics

Characteristic	No. (%)					
	Non-Hispanic White participants (n = 27 974)		Non-Hispanic Black participants (n = 4453)		All participants (n = 32 427)	
	Males	Females	Males	Females	Males	Females
No. of participants	12 265 (42.93)	15 709 (56.16)	1155 (25.94)	3298 (74.06)	13 420 (41.39)	19 007 (58.61)
Age at first cognitive visit, mean (SD), y	74.86 (7.72)	74.95 (8.20)	73.25 (7.28)	73.66 (7.47)	74.72 (7.70)	74.73 (8.09)
Education, mean (SD), y	16.29 (3.10)	15.34 (2.90)	14.29 (3.58)	14.32 (3.17)	16.12 (3.19)	15.16 (2.97)
Clinical diagnosis at first cognitive visit						
Normal cognition	6119 (49.89)	10 024 (63.81)	594 (51.43)	2000 (60.64)	6713 (50.02)	12 024 (63.26)
Impaired	6146 (50.11)	5685 (36.19)	561 (48.57)	1298 (39.36)	6707 (49.98)	6983 (26.74)
*APOE* ε2 count						
0 ε2 Alleles	10 731 (87.49)	13 664 (86.98)	944 (81.73)	2690 (81.56)	11 675 (87.00)	16 354 (86.04)
1 ε2 Allele	1482 (12.08)	1983 (12.62)	198 (17.14)	585 (17.74)	1680 (12.52)	2568 (13.51)
2 ε2 Alleles	52 (0.42)	62 (0.39)	13 (1.13)	23 (0.70)	65 (0.48)	85 (0.45)
*APOE* ε4 count						
0 ε4 Alleles	7580 (61.80)	9936 (63.25)	626 (54.20)	1927 (58.43)	8206 (61.15)	11 863 (62.41)
1 ε4 Allele	3882 (31.65)	4949 (31.50)	449 (38.87)	1175 (35.63)	4331 (32.27)	6124 (32.22)
2 ε4 Alleles	803 (6.55)	824 (5.25)	80 (6.93)	196 (5.94)	883 (6.58)	1020 (5.37)
No. of visits, mean (SD)	4.51 (3.44)	4.97 (3.86)	4.28 (3.26)	4.68 (3.67)	4.49 (3.42)	4.92 (3.83)
Follow-up, mean (SD), y	3.67 (4.20)	3.93 (4.26)	3.38 (3.80)	3.43 (3.79)	3.64 (3.89)	4.13 (4.19)
Memory score, mean (SD)	0.20 (0.81)	0.39 (0.86)	0.07 (0.74)	0.25 (0.77)	0.19 (0.80)	0.37 (0.86)
Executive function score, mean (SD)	0.23 (0.70)	0.34 (0.69)	−0.14 (0.70)	−0.05 (0.68)	0.20 (0.71)	0.27 (0.70)
Language score, mean (SD)	0.28 (0.73)	0.44 (0.78)	0.07 (0.66)	0.15 (0.66)	0.26 (0.73)	0.39 (0.77)

### Main Effects of *APOE* on Cognition

Results of all models are presented in eTable 2 (all diagnoses) and eTable 3 (cognitively normal) in Supplement 2. Among all participants, *APOE *ε4 was robustly associated with worse cognitive performance and faster rates of decline across all cognitive domains, and *APOE *ε2 was associated with better cognitive performance and slower rates of decline. Associations with memory had the largest effects. Results were similar when restricted to participants who were cognitively unimpaired at baseline, although the baseline *APOE *ε2 associations were attenuated.

### Intersectional Effects of Sex, Race, and *APOE* on Cognition

To assess whether the modifying effect of sex on *APOE* differed by race, we performed 3-way interactions of race, sex, and *APOE* (Table 2). One statistically significant intersectional effect was observed among cognitively normal participants, whereby *APOE *ε2 interacted with race and sex on baseline executive function (β = −0.165, SE = 0.066; uncorrected *P* = .01; FDR-corrected *P* = 4.48 × 10^-2^). *APOE *ε2 showed a female-specific protective effect among White participants but a male-specific protective effect among Black participants (Figure, B). A comparable intersectional effect was also observed for *APOE *ε4 on executive function (Figure, A), although this interaction did not survive correction for multiple comparisons. As no other significant intersectional effects of race and sex were observed, the remaining results focus on findings across racial groups.

**Table 2.** Sex × *APOE* × Race Interactions With Multiple Cognitive Domains

Outcome (diagnosis)	*APOE* allele	Sex × *APOE* × race interaction	
		β (SE)	*P* value
**Baseline results in participants with all diagnoses**			
Baseline memory (all diagnoses)	ε2	−0.068 (0.077)	.38
	ε4	0.018 (0.046)	.69
Baseline executive function (all diagnoses)	ε2	−0.132 (0.064)	.04^a^
	ε4	0.018 (0.039)	.65
Baseline language (all diagnoses)	ε2	−0.130 (0.067)	.05
	ε4	0.018 (0.041)	.67
**Baseline results in participants with normal cognition at baseline**			
Baseline memory (normal cognition)	ε2	−0.026 (0.060)	.66
	ε4	0.052 (0.044)	.24
Baseline executive function (normal cognition)	ε2	−0.165 (0.066)	.01^a^^,^^b^
	ε4	0.101 (0.048)	.03^a^
Baseline language (normal cognition)	ε2	−0.104 (0.066)	.12
	ε4	−0.104 (0.052)	.28
**Longitudinal results in participants with all diagnoses**			
Longitudinal memory (all diagnoses)	ε2	−0.007 (0.014)	.61
	ε4	−0.005 (0.009)	.61
Longitudinal executive function (all diagnoses)	ε2	0.009 (0.011)	.45
	ε4	0.005 (0.007)	.46
Longitudinal language (all diagnoses)	ε2	0.003 (0.012)	.83
	ε4	−0.002 (0.008)	.84
**Longitudinal results in participants with normal cognition at baseline**			
Longitudinal memory (normal cognition)	ε2	0.009 (0.014)	.50
	ε4	0.002 (0.010)	.86
Longitudinal executive function (normal cognition)	ε2	0.011 (0.010)	.29
	ε4	0.009 (0.008)	.23
Longitudinal language (normal cognition)	ε2	0.007 (0.011)	.55
	ε4	0.003 (0.008)	.72

^a^ Association had uncorrected *P* < .05.

^b^ Association had false discovery rate–corrected *P* < .05.

**Figure.**
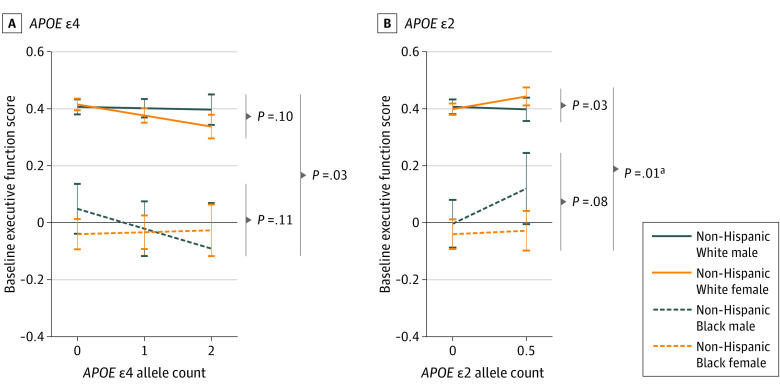
*APOE* × Sex × Race Interactions on Executive Function Performance at Baseline Among Participants With Normal Cognition Sex- and race-specific associations between baseline executive function among cognitively unimpaired individuals in *APOE* ε4 and *APOE* ε2. *P* values are presented for both race-stratified sex × *APOE* interactions and intersectional *APOE* × sex × race interactions. *APOE* ε4 was coded additively, and *APOE* ε2 was coded dominantly. ^a^False discovery rate–corrected *P* < .05.

### Sex Differences in *APOE *Effects by Allele

Among all participants, we observed a stronger association between *APOE *ε4 and baseline memory performance and baseline language performance among women compared with men (although the sex interaction on language was not FDR-significant) (Table 3). For *APOE *ε4, we observed a robust sex interaction on baseline memory (β = −0.071, SE = 0.014; uncorrected *P* = 9.6 × 10^−7^; FDR-corrected *P* = 1.87 × 10^-6^), whereby the *APOE *ε4 negative effect was stronger in females compared with males and did not significantly differ among races. Results were similar across sensitivity analyses. There were no significant sex differences in *APOE *ε4 associations with any of the longitudinal outcomes or when restricting to cognitively normal participants. Among all participants, *APOE *ε2 did not interact with sex on cognition (Table 3).

**Table 3.** Sex- and Race-Specific *APOE* Associations With Multiple Cognitive Domains

Outcome (diagnosis)	*APOE* allele	Model	Cross-ancestry		Non-Hispanic White		Non-Hispanic Black	
			β (SE)	*P* value	β (SE)	*P* value	β (SE)	*P* value
**Baseline results in participants with all diagnoses**								
Baseline memory (all diagnoses)	ε2	Sex-interaction	0.011 (0.027)	.67	0.033 (0.030)	.26	−0.032 (0.063)	.61
		Females	0.215 (0.017)	2.19 × 10^-35^^a^^,^^b^	0.247 (0.020)	4.18 × 10^-35^^a^^,^^b^	0.154 (0.032)	1.93 × 10^-6^^a^^,^^b^
		Males	0.201 (0.020)	1.71 × 10^-23^^a^^,^^b^	0.210 (0.022)	1.63 × 10^-22^^a^^,^^b^	0.187 (0.053)	4.61 × 10^-4^^a^^,^^b^
	ε4	Sex-interaction	−0.071 (0.014)	9.60 × 10^-7^^a^^,^^b^	−0.083 (0.016)	1.40 × 10^-7^^a^^,^^b^	−0.066 (0.038)	.08
		Females	−0.422 (0.010)	<2.23 × 10^-308^^a^^,^^b^	−0.441 (0.011)	<2.23 × 10^-308^^a^^,^^b^	−0.316 (0.020)	1.81 × 10^-54^^a^^,^^b^
		Males	−0.341 (0.011)	7.68 × 10^-217^^a^^,^^b^	−0.347 (0.011)	1.95 × 10^-202^^a^^,^^b^	−0.247 (0.032)	4.63 × 10^-14^^a^^,^^b^
Baseline executive function (all diagnoses)	ε2	Sex-interaction	0.002 (0.023)	.98	0.036 (0.024)	.15	−0.094 (0.057)	.10
		Females	0.083 (0.014)	6.85 × 10^-9^^a^^,^^b^	0.126 (0.016)	1.23 × 10^-15^^a^^,^^b^	0.062 (0.029)	.03^a^^,^^b^
		Males	0.079 (0.018)	1.18 × 10^-5^^a^^,^^b^	0.088 (0.019)	3.65 × 10^-6^^a^^,^^b^	0.157 (0.051)	2.24 × 10^-3^^a^^,^^b^
	ε4	Sex-interaction	−0.009 (0.013)	.49	−0.015 (0.013)	.26	0.000 (0.036)	.99
		Females	−0.200 (0.008)	3.59 × 10^-124^^a^^,^^b^	−0.205 (0.009)	4.65 × 10^-113^^a^^,^^b^	−0.131 (0.019)	1.74 × 10^-12^^a^^,^^b^
		Males	−0.018 (0.010)	6.20 × 10^-77^^a^^,^^b^	−0.182 (0.010)	1.23 × 10^-70^^a^^,^^b^	−0.129 (0.032)	5.50 × 10^-5^^a^^,^^b^
Baseline language (all diagnoses)	ε2	Sex-interaction	−0.023 (0.024)	.34	0.009 (0.026)	.74	−0.121 (0.055)	.03^a^^,^^b^
		Females	0.095 (0.015)	3.38 × 10^-10^^a^^,^^b^	0.129 (0.017)	1.10 × 10^-13^^a^^,^^b^	0.080 (0.028)	4.01 × 10^-3^^a^^,^^b^
		Males	0.114 (0.018)	2.54 × 10^-10^^a^^,^^b^	0.114 (0.019)	2.84 × 10^-9^^a^^,^^b^	0.201 (0.047)	2.15 × 10^-5^^a^^,^^b^
	ε4	Sex-interaction	−0.027 (0.013)	.04^a^	−0.031 (0.014)	.03^a^^,^^b^	−0.014 (0.034)	.69
		Females	−0.216 (0.009)	1.68 × 10^-131^^a^^,^^b^	−0.220 (0.010)	1.35 × 10^-109^^a^^,^^b^	−0.172 (0.018)	4.19 × 10^-22^^a^^,^^b^
		Males	−0.176 (0.010)	6.87 × 10^-72^^a^^,^^b^	−0.174 (0.010)	1.29 × 10^-63^^a^^,^^b^	−0.156 (0.029)	9.53 × 10^-8^^a^^,^^b^
**Baseline results in participants with normal cognition at baseline**								
Baseline memory (normal cognition)	ε2	Sex-interaction	−0.015 (0.021)	.49	−0.004 (0.002)	.85	−0.031 (0.052)	.56
		Females	−0.007 (0.012)	.60	0.014 (0.014)	.32	−0.004 (0.025)	.88
		Males	0.006 (0.017)	.71	0.015 (0.017)	.40	0.026 (0.045)	.56
	ε4	Sex-interaction	−0.016 (0.015)	.27	−0.024 (0.016)	.13	0.028 (0.038)	.47
		Females	−0.049 (0.009)	6.59 × 10^-8^^a^^,^^b^	−0.046 (0.010)	3.49 × 10^-6^^a^^,^^b^	−0.020 (0.019)	.30
		Males	−0.025 (0.012)	.03^a^	−0.013 (0.012)	.30	−0.049 (0.032)	.13
Baseline executive function (normal cognition)	ε2	Sex-interaction	0.021 (0.023)	.38	0.052 (0.024)	.03^a^	−0.112 (0.064)	.08
		Females	0.011 (0.014)	.44	0.045 (0.015)	2.29 × 10^-3^^a^^,^^b^	0.012 (0.030)	.68
		Males	−0.011 (0.019)	.56	−0.009 (0.019)	.64	0.124 (0.060)	.04^a^
	ε4	Sex-interaction	−0.013 (0.017)	.44	−0.028 (0.017)	.10	0.074 (0.046)	.11
		Females	−0.044 (0.010)	1.16 × 10^-5^^a^^,^^b^	−0.039 (0.011)	2.20 × 10^-4^^a^^,^^b^	0.007 (0.002)	.76
		Males	−0.027 (0.013)	.04^a^	−0.005 (0.014)	.72	−0.070 (0.043)	.11
Baseline language (normal cognition)	ε2	Sex-interaction	−0.036 (0.023)	.12	−0.012 (0.025)	.63	−0.117 (0.057)	.04^a^
		Females	−0.029 (0.014)	.04^a^	−0.003 (0.015)	.85	−0.008 (0.027)	.77
		Males	0.003 (0.018)	.88	0.002 (0.019)	.92	0.109 (0.051)	.03^a^
	ε4	Sex-interaction	−0.001 (0.016)	.95	−0.002 (0.018)	.91	0.050 (0.041)	.23
		Females	−0.017 (0.010)	.10	−0.002 (0.011)	.86	−0.021 (0.021)	.31
		Males	−0.001 (0.013)	.94	0.019 (0.018)	.17	−0.069 (0.036)	.06
**Longitudinal results in participants with all diagnoses**								
Longitudinal memory (all diagnoses)	ε2	Sex-interaction	−0.004 (0.005)	.42	−0.003 (0.005)	.58	−0.010 (0.011)	.34
		Females	0.029 (0.003)	7.54 × 10^-24^^a^^,^^b^	0.030 (0.003)	1.35 × 10^-18^^a^^,^^b^	0.020 (0.005)	1.36 × 10^-4^^a^^,^^b^
		Males	0.036 (0.004)	9.96 × 10^-19^^a^^,^^b^	0.035 (0.004)	1.11 × 10^-15^^a^^,^^b^	0.032 (0.010)	1.34 × 10^-3^^a^^,^^b^
	ε4	Sex-interaction	0.006 (0.003)	.046^a^	0.004 (0.003)	.21	0.000 (0.007)	.96
		Females	−0.054 (0.002)	7.02 × 10^-190^^a^^,^^b^	−0.060 (0.002)	6.47 × 10^-177^^a^^,^^b^	−0.037 (0.004)	1.13 × 10^-24^^a^^,^^b^
		Males	−0.062 (0.002)	2.08 × 10^-157^^a^^,^^b^	−0.065 (0.003)	2.82 × 10^-154^^a^^,^^b^	−0.039 (0.006)	7.03 × 10^-10^^a^^,^^b^
Longitudinal executive function (all diagnoses)	ε2	Sex-interaction	−0.004 (0.004)	.30	−0.005 (0.004)	.23	0.004 (0.009)	.68
		Females	0.016 (0.002)	4.17 × 10^-12^^a^^,^^b^	0.015 (0.003)	6.88 × 10^-9^^a^^,^^b^	0.014 (0.004)	6.17 × 10^-4^^a^^,^^b^
		Males	0.021 (0.003)	2.72 × 10^-11^^a^^,^^b^	0.022 (0.003)	1.68 × 10^-10^^a^^,^^b^	0.011 (0.008)	.16
	ε4	Sex-interaction	0.004 (0.002)	.07	0.002 (0.003)	.47	0.008 (0.006)	.16
		Females	−0.032 (0.002)	5.97 × 10^-104^^a^^,^^b^	−0.037 (0.002)	1.99 × 10^-105^^a^^,^^b^	−0.016 (0.003)	6.09 × 10^-8^^a^^,^^b^
		Males	−0.038 (0.002)	6.88 × 10^-90^^a^^,^^b^	−0.041 (0.002)	1.61 × 10^-88^^a^^,^^b^	−0.024 (0.005)	2.39 × 10^-6^^a^^,^^b^
Longitudinal language (all diagnoses)	ε2	Sex-interaction	−0.007 (0.004)	.10	−0.007 (0.005)	.13	−0.004 (0.009)	.62
		Females	0.022 (0.003)	2.43 × 10^-17^^a^^,^^b^	0.022 (0.003)	2.61 × 10^-13^^a^^,^^b^	0.014 (0.005)	1.50 × 10^-3^^a^^,^^b^
		Males	0.030 (0.003)	2.00 × 10^-18^^a^^,^^b^	0.030 (0.004)	7.71 × 10^-16^^a^^,^^b^	0.019 (0.008)	.01^a^^,^^b^
	ε4	Sex-interaction	0.004 (0.003)	.14	0.002 (0.003)	.58	0.002 (0.006)	.79
		Females	−0.042 (0.002)	8.52 × 10^-148^^a^^,^^b^	−0.048 (0.002)	7.09 × 10^-147^^a^^,^^b^	−0.023 (0.003)	6.55 × 10^-13^^a^^,^^b^
		Males	−0.047 (0.002)	2.64 × 10^-124^^a^^,^^b^	−0.051 (0.002)	2.17 × 10^-124^^a^^,^^b^	−0.025 (0.005)	5.98 × 10^-7^^a^^,^^b^
**Longitudinal results in participants with normal cognition at baseline**								
Longitudinal memory (normal cognition)	ε2	Sex-interaction	−0.002 (0.005)	.67	−0.004 (0.005)	.42	0.004 (0.011)	.69
		Females	0.010 (0.003)	2.62 × 10^-4^^a^^,^^b^	0.008 (0.003)	.01^a^^,^^b^	0.014 (0.005)	.01^a^^,^^b^
		Males	0.012 (0.004)	6.20 × 10^-4^^a^^,^^b^	0.012 (0.004)	1.56 × 10^-3^^a^^,^^b^	0.010 (0.009)	.29
	ε4	Sex-interaction	−0.001 (0.003)	.89	−0.002 (0.004)	.66	0.000 (0.008)	.96
		Females	−0.024 (0.002)	8.52 × 10^-33^^a^^,^^b^	−0.027 (0.002)	3.16 × 10^-32^^a^^,^^b^	−0.014 (0.004)	5.13 × 10^-4^^a^^,^^b^
		Males	−0.022 (0.003)	1.80 × 10^-18^^a^^,^^b^	−0.024 (0.003)	2.34 × 10^-18^^a^^,^^b^	−0.013 (0.007)	.05
Longitudinal executive function (normal cognition)	ε2	Sex-interaction	−0.001 (0.003)	.76	−0.003 (0.004)	.43	0.008 (0.009)	.36
		Females	0.006 (0.002)	2.20 × 10^-3^^a^^,^^b^	0.005 (0.002)	.045^a^	0.011 (0.004)	.01^a^^,^^b^
		Males	0.007 (0.003)	7.76 × 10^-3^^a^^,^^b^	0.008 (0.003)	8.61 × 10^-3^^a^^,^^b^	0.003 (0.008)	.72
	ε4	Sex-interaction	−0.001 (0.003)	.76	−0.002 (0.003)	.37	0.007 (0.007)	.34
		Females	−0.013 (0.002)	2.18 × 10^-19^^a^^,^^b^	−0.015 (0.002)	5.86 × 10^-21^^a^^,^^b^	−0.005 (0.003)	.17
		Males	−0.013 (0.002)	1.13 × 10^-10^^a^^,^^b^	−0.013 (0.002)	3.44 × 10^-10^^a^^,^^b^	−0.010 (0.006)	.06
Longitudinal language (normal cognition)	ε2	Sex-interaction	−0.005 (0.004)	.15	−0.007 (0.004)	.09	−0.001 (0.009)	.88
		Females	0.006 (0.002)	4.74 × 10^-3^^a^^,^^b^	0.005 (0.003)	.08	0.009 (0.004)	.04^a^
		Males	0.012 (0.003)	3.12 × 10^-5^^a^^,^^b^	0.011 (0.003)	1.57 × 10^-4^^a^^,^^b^	0.010 (0.007)	.15
	ε4	Sex-interaction	−0.003 (0.003)	.31	−0.004 (0.003)	.15	−0.002 (0.007)	.78
		Females	−0.016 (0.002)	3.15 × 10^-23^^a^^,^^b^	−0.020 (0.002)	9.84 × 10^-27^^a^^,^^b^	−0.004 (0.004)	.24
		Males	−0.013 (0.002)	1.50 × 10^-10^^a^^,^^b^	−0.015 (0.002)	1.40 × 10^-11^^a^^,^^b^	−0.003 (0.005)	.57

^a^ Association had uncorrected *P* < .05.

^b^ Association had false discovery rate–corrected *P* < .05.

## Discussion

To our knowledge, this study is the largest investigation of the modifying effects of both sex and race on the association between *APOE* and AD-related cognitive decline. We provide strong evidence of a sex difference in the association between *APOE *ε4 and cross-sectional memory performance and, for the first time, also extend this sex difference to the language domain. Furthermore, these *APOE *ε4 effects were stronger in females, and there was no statistical evidence of race specificity. In contrast to *APOE *ε4, we did not observe evidence of sex differences in *APOE *ε2 associations with cognition across race. However, we did observe exciting novel evidence of intersectional effects for *APOE *ε2, whereby sex-specific associations on cognition may differ by racial group, particularly in the executive function domain. Together, our findings solidify current understandings of sex-specific effects of *APOE *ε4 while highlighting the pressing need to fully characterize intersectional effects of race, sex, and *APOE *ε2 on late-life cognition.

The strong association between *APOE *ε4 and cognition among women aligns with and extends past work. *APOE *ε4 showed stronger effects on baseline memory and language performance among females compared with males, and this effect was similar among Black and White individuals. Interestingly, these female-driven effects of *APOE *ε4 are only present when evaluating across clinical diagnoses and not among the cognitively unimpaired subset. Indeed, *APOE *ε4 is a stronger predictor of MCI and clinical AD risk among females compared with males, particularly among those aged 55 to 70 years.^10,16^ Likewise, past work has observed female ε4 carriers to have faster memory decline compared with male ε4 carriers.^8^ That said, there has not been much characterization of sex differences in *APOE *ε4 effects on language, making our findings of increased ε4 effects among females interesting. The mechanism underlying these female-specific findings, in both our research and that of other groups, remains unclear. Sex hormones may play a role, as studies have demonstrated estrogen levels affect cognition.^50,51,52^ Menopausal loss of estrogen may amplify negative *APOE *ε4 effects, resulting in greater cognitive impairment compared with males. Additionally, females tend to have steeper cognitive decline in the presence of high levels of AD neuropathology^53^; therefore, the effects of ε4 certainly could be driven by more advanced neuropathology. Tau pathology has been implicated as a potential pathway previously, as prior studies have highlighted stronger associations between *APOE *ε4 and biomarkers of tau pathology in females compared with males.^7,54,55^ Regardless of the underlying mechanism, the present results solidify the evidence of robust sex differences in the risk effect of *APOE *ε4.

Given the lower allele frequency and smaller effect size of ε2, few studies have explored sex- and race-specific *APOE *ε2 effects on cognitive decline. Given our large sample size, we were able to provide strong evidence that the sex difference in *APOE* ε4 risk does not extend to *APOE *ε2 protection, with strikingly consistent associations between *APOE *ε2 and cognition in males and females. Fascinatingly, among cognitive normal participants we do provide exciting new evidence of an intersectional effect of sex, race, and *APOE *ε2 on executive function. More specifically, the *APOE *ε2 protective effect was female-specific in White individuals but male-specific in Black individuals. Very few studies have evaluated such intersectional effects of *APOE *ε2 on cognition. In a study of 976 African American and 794 White middle-aged adults, Beydoun and colleagues^22^ investigated sex and race as potential modifiers of *APOE* on cognition. While the investigators noted a lack of consistency in associations between *APOE *ε2 and specific neuropsychological test scores across races, no race- or sex-specific associations were deemed significant.^22^ Studies are also mixed regarding differential *APOE *ε2 effects among the sexes, with one study demonstrating stronger effects among cognitively normal males,^7^ but another highlighting stronger effects among females.^14^ Additionally, a recent study found that the *APOE *ε2 association with cognitive decline in cognitively normal White men was stronger than in White women.^56^ In the present analysis, which included some of the same cohorts, we saw similar evidence of a stronger association between *APOE *ε2 and longitudinal decline in cognitively normal White men compared with cognitively normal White women, but the *APOE *ε2 × sex interaction did not reach statistical significance (Table 3). Continued reporting by sex and race will allow for larger studies to confirm whether the intersectional effects and the previous mixed findings in *APOE *ε2 carriers persist.

Because *APOE *ε2 and ε4 are known to have opposing effects, we performed sensitivity analyses excluding participants with the ε2/ε4 genotype. In our analyses, sex × *APOE* effects on cognition were generally strengthened after ε2/ε4 genotype removal. These results are in line with previous studies, which have reported as much as a 3-fold increase in the odds of developing AD for those with the ε2/ε4 genotype.^57,58,59,60^ We also conducted age-stratified sensitivity analyses to determine if significant sex × *APOE* interactions on cognition differed between participants younger than 75 years and participants 75 years or older at baseline. Consistent with past literature,^10^ we observed that sex × *APOE *ε4 interactions on cognition were primarily driven by the younger age group. In contrast to ε4, our results suggest that sex may modify the association between *APOE *ε2 and cognition among older participants. A previous study showed *APOE *ε2 protective effects against clinical dementia in the oldest old (≥90 years), so this will be an exciting avenue for future work.

### Strengths and Limitations

The present study has several strengths. Longitudinal, harmonized cognitive data across multiple cognitive domains facilitated a large sample size, which allowed for investigation of the intersectional effects of race, sex, and *APOE* on cognition. However, our results should be interpreted taking weaknesses into consideration. While sex and race effects of amyloid-β and tau pathologies are important to consider in the context of *APOE* and cognition, harmonized biomarker data were not readily available; therefore, we could not integrate neuropathology and biomarker measures into our models. Additionally, only data for Black and White individuals were included in this study, so our results may not be generalizable to those in other racial and ethnic groups. Moreover, despite the large sample size, the number of ε2 homozygotes was small and warrants further exploration. To maximize statistical power in sex/race stratifications, *APOE *ε2 was modeled dominantly. Results from dominant and additive *APOE *ε2 models were, in general, similar (eTables 2 and 3 in Supplement 2); however, prior literature indicates White individuals with an *APOE *ε2/ε3 genotype have a significantly lower risk of AD compared with individuals with the *APOE *ε2/ε2 genotype.^6^ Whether this holds true among Black individuals is unclear, again highlighting the need for even larger sample sizes of racially diverse individuals.

Additionally, though this study leveraged harmonized cognitive scores from multiple cohorts, we observed some heterogeneity of effects across cohorts, as seen in eFigures 1 and 2 in Supplement 1. Most heterogeneity is likely driven by sample size differences, with the largest cohort (NACC) often driving significant results. Furthermore, while cohorts were of similar age and highly educated, recruitment schemes differed. Therefore, the presence of associations may be due to the relative frequency of AD cases and selection bias differences across study designs. The majority of our significant associations were observed when including all participants regardless of clinical diagnosis and thus are more likely reflective of the clinic-based sampling cohort from NACC. Moreover, *APOE *ε4 effects have been shown to vary with genetic ancestry, especially within African American subpopulations.^61^ While covarying for ancestry principal components did not affect our interpretation of results, evaluation of local ancestry at the *APOE* locus would allow for better dissociation of population effects.

## Conclusion

The results of our study confirmed sex differences in the association between *APOE *ε4 and memory and provide strong evidence this sex difference does not differ among Black and White individuals. In contrast, we did not see robust sex differences in *APOE *ε2 effects on cognition, though we provide exciting new evidence of an intersectional effect of sex, race, and *APOE *ε2 on cognition. Our finding that *APOE* isoforms were differently modified by sex and race highlights the fact that ε2 and ε4 are not simply opposing sides of the same coin and underscores the need for a comprehensive precision-medicine approach in understanding AD progression.

## Notes

Supplement 1. **eFigure 1.** Forest plots of significant APOE-ε4*sex interactions **eFigure 2.** Forest plots of significant APOE-ε2*sex interactions

Supplement 2. **eTable 1.** Demographics by Cohort **eTable 2.** APOE x Race x Sex Interactions on Cognition (All Diagnoses) **eTable 3.** APOE x Race x Sex Interactions on Cognition (Normal Cognition)

Supplement 3. Nonauthor collaborators

Supplement 4. Data sharing statement
