# Supplementary material for: Associations of Sex, Race, and Apolipoprotein E Alleles With Multiple Domains of Cognition Among Older Adults
Source: JAMA Neurol. 2023 Jul 17;80(9):929–39. doi: 10.1001/jamaneurol.2023.2169 (PMC10352930; doi:10.1001/jamaneurol.2023.2169)
Supplement: Supplement 1. — eFigure 1. Forest plots of significant APOE-ε4*sex interactions eFigure 2. Forest plots of significant APOE-ε2*sex interactions [file jamaneurol-e232169-s001.pdf]

Supplemental Online Content

Walters S, Contreras AG, Eissman JM, et al; Alzheimer's Disease Neuroimaging Initiative, Alzheimer's Disease Genetics Consortium, and Alzheimer's Disease Sequencing Project. Associations of sex, race, and apolipoprotein E alleles with multiple domains of cognition among older adults. *JAMA Neurol*. Published online July 17, 2023. doi:10.1001/jamaneurol.2023.2169

eFigure 1. Forest plots of significant APOE-ε4*sex interactions

eFigure 2. Forest plots of significant APOE-ε2*sex interactions

This supplemental material has been provided by the authors to give readers additional information about their work.

© 2023 Walters S et al. *JAMA Neurology.*

eFigure 1. Forest plots of significant *APOE*-ε4*sex interactions

For the purpose of comparison, individual cohort-level results are provided for associations with *APOE*-ε4 that show evidence of significance. ADNI=the Alzheimer's Disease Neuroimaging Initiative; ROSMAP=the three harmonized cohorts of the Religious Orders Study, Rush Memory and Aging Project, and Minority Aging Research Study; NACC=the National Alzheimer's Coordinating Center; ACT=Adult Changes in Thought; NHW=non-Hispanic Whites; NC=normal cognition

A. All races, baseline memory~sex*APOE-ε4 (all diagnoses)

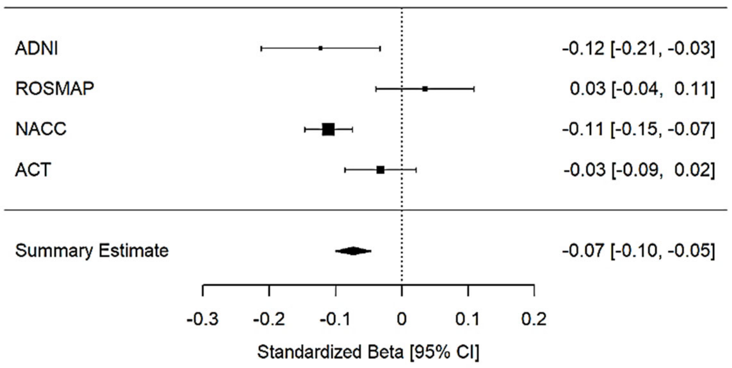

B. All races, baseline language~sex*APOE-ε4 (all diagnoses)

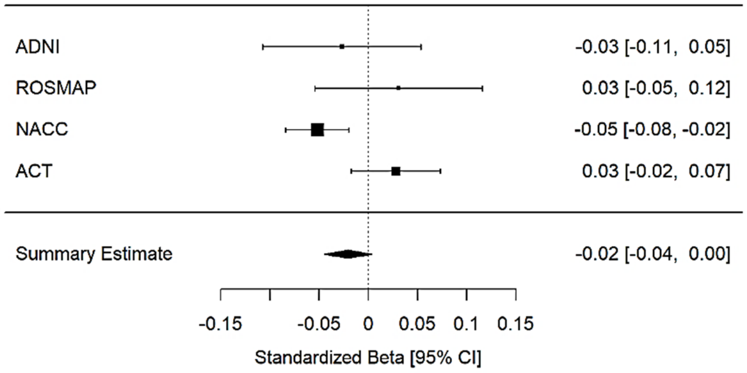

C. All races, baseline executive function~sex*APOE-ε4*race (NC)

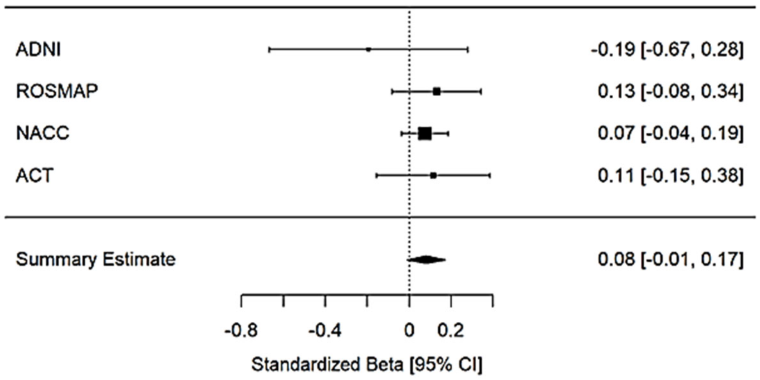

© 2023 Walters S et al. *JAMA Neurology.*

eFigure 2. Forest plots for significant *APOE*-ε2*sex interactions

For the purpose of comparison, individual cohort-level results are provided for associations with *APOE*-ε2 that show evidence of significance. ADNI=the Alzheimer's Disease Neuroimaging Initiative; ROSMAP=the three harmonized cohorts of the Religious Orders Study, Rush Memory and Aging Project, and Minority Aging Research Study; NACC=the National Alzheimer's Coordinating Center; ACT=Adult Changes in Thought; NHW=non-Hispanic Whites; NHB=non-Hispanic Blacks; NC=normal cognition

A. All races, baseline executive function~sex**APOE*-ε2*race (NC)

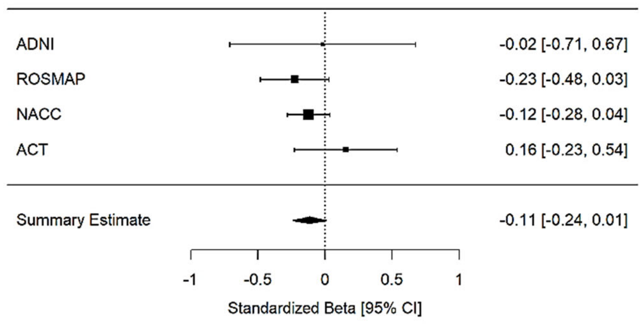

© 2023 Walters S et al. *JAMA Neurology.*
