# Supplementary material for: Associations of Sex, Race, and Apolipoprotein E Alleles With Multiple Domains of Cognition Among Older Adults
Source: JAMA Neurol. 2023 Jul 17;80(9):929–39. doi: 10.1001/jamaneurol.2023.2169 (PMC10352930; doi:10.1001/jamaneurol.2023.2169)
Supplement: Supplement 3. — Nonauthor collaborators [file jamaneurol-e232169-s003.pdf]

Supplemental Online Content: Nonauthor Collaborators
*First name, last name, and suffix (if applicable) are required and will appear in PubMed.

*Group Name(s): the Alzheimer's Disease Genetics Consortium (ADCG), the Alzheimer's Disease Neuroimaging Initiative (ADNI), the Alzheimer's Disease Sequencing Project (ADSP)							
***First Name and Middle Initial(s)**	***Last Name**	***Suffix (eg, Jr, III)**	Academic Degrees	Institution	Location (city, state/province, country)	Role or Contribution, eg, chair, principal investigator	Group (if more than 1 Group listed in the byline) and/or Subgroup (eg, Steering Committee)
Erin	Abner		PhD	University of Kentucky			Alzheimer's Disease Genetics Consortium
Perrie	Adams		PhD	University of Texas Southwestern Medical Center			Alzheimer's Disease Genetics Consortium
Alyssa	Aguirre		LCSW	University of Texas at Austin/Dell Medical School			Alzheimer's Disease Genetics Consortium
Marilyn	Albert		PhD	Johns Hopkins University			Alzheimer's Disease Genetics Consortium
Roger	Albin		MD	University of Michigan			Alzheimer's Disease Genetics Consortium
Mariet	Allen		PhD	Mayo Clinic			Alzheimer's Disease Genetics Consortium
Lisa	Alvarez			University of North Texas Health Science Center			Alzheimer's Disease Genetics Consortium
Liana	Apostolova		MD	Indiana University			Alzheimer's Disease Genetics Consortium
Steven	Arnold		MD	University of Pennsylvania Perelman School of Medicine			Alzheimer's Disease Genetics Consortium
Sanjay	Asthana		MD	University of Wisconsin			Alzheimer's Disease Genetics Consortium
Craig	Atwood		PhD	University of Wisconsin			Alzheimer's Disease Genetics Consortium
Gayle	Ayres		DO	University of Texas at Austin/Dell Medical School			Alzheimer's Disease Genetics Consortium
Robert	Barber		PhD	University of North Texas Health Science Center			Alzheimer's Disease Genetics Consortium
Lisa	Barnes		PhD	Rush University Medical Center			Alzheimer's Disease Genetics Consortium

Supplemental Online Content: Nonauthor Collaborators
*First name, last name, and suffix (if applicable) are required and will appear in PubMed.

***First Name and Middle Initial(s)**	***Last Name**	***Suffix (eg, Jr, III)**	Academic Degrees	Institution	Location (city, state/province, country)	Role or Contribution, eg, chair, principal investigator	Group (if more than 1 Group listed in the byline) and/or Subgroup (eg, Steering Committee)
Sandra	Barral		PhD	Columbia University			Alzheimer's Disease Genetics Consortium
Jackie	Bartlett		PhD	Case Western Reserve University			Alzheimer's Disease Genetics Consortium
Thomas	Beach		MD, PhD	Banner Sun Health Research Institute			Alzheimer's Disease Genetics Consortium
James	Becker		PhD	University of Pittsburgh School of Medicine			Alzheimer's Disease Genetics Consortium
Gary	Beecham		PhD	University of Miami			Alzheimer's Disease Genetics Consortium
Penelope	Benchek		PhD	Case Western Reserve University			Alzheimer's Disease Genetics Consortium
David	Bennett		MD	Rush University Medical Center			Alzheimer's Disease Genetics Consortium
John	Bertelson		MD	University of Texas at Austin/Dell Medical School			Alzheimer's Disease Genetics Consortium
Sarah	Biber		PhD	University of Washington			Alzheimer's Disease Genetics Consortium
Thomas	Bird		MD	University of Washington			Alzheimer's Disease Genetics Consortium
Deborah	Blacker		MD	Harvard School of Public Health			Alzheimer's Disease Genetics Consortium
Bradley	Boeve		MD	Mayo Clinic			Alzheimer's Disease Genetics Consortium
James	Bowen		MD	Swedish Medical Center			Alzheimer's Disease Genetics Consortium
Adam	Boxer		MD, PhD	University of California San Francisco			Alzheimer's Disease Genetics Consortium
James	Brewer		MD	University of California San Diego			Alzheimer's Disease Genetics Consortium

*First name, last name, and suffix (if applicable) are required and will appear in PubMed.

***First Name and Middle Initial(s)**	***Last Name**	***Suffix (eg, Jr, III)**	Academic Degrees	Institution	Location (city, state/province, country)	Role or Contribution, eg, chair, principal investigator	Group (if more than 1 Group listed in the byline) and/or Subgroup (eg, Steering Committee)
James	Burke		MD, PhD	Duke University			Alzheimer's Disease Genetics Consortium
Jeffery	Burns		MD, MS	University of Kansas Medical Center			Alzheimer's Disease Genetics Consortium
William	Bush		PhD	Case Western Reserve University			Alzheimer's Disease Genetics Consortium
Joseph	Buxbaum		PhD	Mount Sinai School of Medicine			Alzheimer's Disease Genetics Consortium
Goldie	Byrd		PhD	Wake Forest University			Alzheimer's Disease Genetics Consortium
Laura	Cantwell		MPH	University of Pennsylvania Perelman School of Medicine			Alzheimer's Disease Genetics Consortium
Chuanhai	Cao		PhD	University of South Florida			Alzheimer's Disease Genetics Consortium
Cynthia	Carlsson		MD	University of Wisconsin			Alzheimer's Disease Genetics Consortium
Minerva	Carrasquillo		PhD	Mayo Clinic			Alzheimer's Disease Genetics Consortium
Kwun	Chan		PhD	University of Washington			Alzheimer's Disease Genetics Consortium
Scott	Chase		PhD	University of North Carolina			Alzheimer's Disease Genetics Consortium
Yen-Chi	Chen		PhD	University of Washington			Alzheimer's Disease Genetics Consortium
Marie-Franciose	Chesselet		PhD	University of California Los Angeles			Alzheimer's Disease Genetics Consortium
Nathaniel	Chin		MD	University of Wisconsin			Alzheimer's Disease Genetics Consortium
Helena	Chui		MD	University of Southern California			Alzheimer's Disease Genetics Consortium

Supplemental Online Content: Nonauthor Collaborators
*First name, last name, and suffix (if applicable) are required and will appear in PubMed.

***First Name and Middle Initial(s)**	***Last Name**	***Suffix (eg, Jr, III)**	Academic Degrees	Institution	Location (city, state/province, country)	Role or Contribution, eg, chair, principal investigator	Group (if more than 1 Group listed in the byline) and/or Subgroup (eg, Steering Committee)
Jaeyoon	Chung		PhD	Boston University			Alzheimer's Disease Genetics Consortium
Suzanne	Craft		PhD	Wake Forest University			Alzheimer's Disease Genetics Consortium
Paul	Crane		MD, MPH	University of Washington			Alzheimer's Disease Genetics Consortium
Carlos	Cruchaga		PhD	Washington University School of Medicine			Alzheimer's Disease Genetics Consortium
Michael	Cuccaro		PhD	University of Miami			Alzheimer's Disease Genetics Consortium
Jessica	Culhane		PhD	University of Washington			Alzheimer's Disease Genetics Consortium
C. Munro	Cullum		PhD	University of Texas Southwestern Medical Center			Alzheimer's Disease Genetics Consortium
Eveleen	Darby		MA, MS	Baylor College of Medicine			Alzheimer's Disease Genetics Consortium
Barbara	Davis		MA, MS	University of Texas Southwestern Medical Center			Alzheimer's Disease Genetics Consortium
Charles	DeCarli		MD	University of California Davis			Alzheimer's Disease Genetics Consortium
John	DeToledo		MD	Texas Tech University Health Science Center			Alzheimer's Disease Genetics Consortium
Dennis	Dickson		MD	Mayo Clinic			Alzheimer's Disease Genetics Consortium
Nic	Dobbins		PhD	University of Washington			Alzheimer's Disease Genetics Consortium
Ranjan	Duara		MD	Mount Sinai Medical Center			Alzheimer's Disease Genetics Consortium
Nilufer	Ertekin-Taner		MD, PhD	Mayo Clinic			Alzheimer's Disease Genetics Consortium

*First name, last name, and suffix (if applicable) are required and will appear in PubMed.

*First Name and Middle Initial(s)	*Last Name	*Suffix (eg, Jr, III)	Academic Degrees	Institution	Location (city, state/province, country)	Role or Contribution, eg, chair, principal investigator	Group (if more than 1 Group listed in the byline) and/or Subgroup (eg, Steering Committee)
Denis	Evans		MD	Rush University Medical Center			Alzheimer's Disease Genetics Consortium
Kelley	Faber		MS	Indiana University			Alzheimer's Disease Genetics Consortium
Thomas	Fairchild		PhD	University of North Texas Health Science Center			Alzheimer's Disease Genetics Consortium
Daniele	Fallin		PhD	Johns Hopkins University			Alzheimer's Disease Genetics Consortium
Kenneth	Fallon		MD	University of Alabama at Birmingham			Alzheimer's Disease Genetics Consortium
David	Fardo		PhD	University of Kentucky			Alzheimer's Disease Genetics Consortium
Martin	Farlow		MD	Indiana University			Alzheimer's Disease Genetics Consortium
John	Farrell		PhD	Boston University			Alzheimer's Disease Genetics Consortium
Lindsay	Farrer		PhD	Boston University			Alzheimer's Disease Genetics Consortium
Victoria	Fernandez-Hernandez			Washington University			Alzheimer's Disease Genetics Consortium
Tatiana	Foroud		PhD	Indiana University			Alzheimer's Disease Genetics Consortium
Matthew	Frosch		PhD	Massachusetts General Hospital			Alzheimer's Disease Genetics Consortium
Douglas	Galasko		MD	University of California San Diego			Alzheimer's Disease Genetics Consortium
Adriana	Gamboa		BS	University of North Texas Health Science Center			Alzheimer's Disease Genetics Consortium
Daniel	Geschwind		MD, PhD	University of California Los Angeles			Alzheimer's Disease Genetics Consortium

Supplemental Online Content: Nonauthor Collaborators
*First name, last name, and suffix (if applicable) are required and will appear in PubMed.

*First Name and Middle Initial(s)	*Last Name	*Suffix (eg, Jr, III)	Academic Degrees	Institution	Location (city, state/province, country)	Role or Contribution, eg, chair, principal investigator	Group (if more than 1 Group listed in the byline) and/or Subgroup (eg, Steering Committee)
Bernadino	Ghetti		MD	Indiana University			Alzheimer's Disease Genetics Consortium
Alison	Goate		D.Phil	Mount Sinai School of Medicine			Alzheimer's Disease Genetics Consortium
Thomas	Grabowski		MD	University of Washington			Alzheimer's Disease Genetics Consortium
Neill	Graff-Radford		MD	Mayo Clinic			Alzheimer's Disease Genetics Consortium
Anthony	Griswold		PhD	University of Miami			Alzheimer's Disease Genetics Consortium
Jonathan	Haines		PhD	Case Western Reserve University			Alzheimer's Disease Genetics Consortium
Hakon	Hakonarson		MD, PhD	Children's Hospital of Philadelphia			Alzheimer's Disease Genetics Consortium
Kathleen	Hall		PhD	Indiana University			Alzheimer's Disease Genetics Consortium
James	Hall		PhD	University of North Texas Health Science Center			Alzheimer's Disease Genetics Consortium
Ronald	Hamilton		MD	University of Pittsburgh			Alzheimer's Disease Genetics Consortium
Kara	Hamilton-Nelson		MPH	University of Miami			Alzheimer's Disease Genetics Consortium
Xudong	Han		PhD	Boston University			Alzheimer's Disease Genetics Consortium
John	Hardy		PhD	University College London			Alzheimer's Disease Genetics Consortium
Lindy	Harrell		MD, PhD	University of Alabama at Birmingham			Alzheimer's Disease Genetics Consortium
Elizabeth	Head		PHD	University of California Irvine			Alzheimer's Disease Genetics Consortium

Supplemental Online Content: Nonauthor Collaborators
*First name, last name, and suffix (if applicable) are required and will appear in PubMed.

*First Name and Middle Initial(s)	*Last Name	*Suffix (eg, Jr, III)	Academic Degrees	Institution	Location (city, state/province, country)	Role or Contribution, eg, chair, principal investigator	Group (if more than 1 Group listed in the byline) and/or Subgroup (eg, Steering Committee)
Victor	Henderson		MD, MS	Stanford University			Alzheimer's Disease Genetics Consortium
Michelle	Hernandez		BS	Texas Tech University Health Science Center			Alzheimer's Disease Genetics Consortium
Lindy	Harrell		MD, PhD	University of Alabama at Birmingham			Alzheimer's Disease Genetics Consortium
Lawrence	Honig		MD, PhD	Columbia University			Alzheimer's Disease Genetics Consortium
Ryan	Huebinger		PhD	University of Texas Southwestern Medical Center			Alzheimer's Disease Genetics Consortium
Matthew	Huentelman		PhD	Translational Genomics Research Institute			Alzheimer's Disease Genetics Consortium
Christine	Hulette		MD	Duke University			Alzheimer's Disease Genetics Consortium
Bradley	Hyman		MD, PhD	Massachusetts General Hospital/Harvard Medical School			Alzheimer's Disease Genetics Consortium
Linda	Hynan		PhD	University of Texas Southwestern Medical Center			Alzheimer's Disease Genetics Consortium
Laura	Ibanez		BS	Washington University			Alzheimer's Disease Genetics Consortium
Philip	De Jager		MD PhD	Brigham and Women's Hospital and Harvard Medical School			Alzheimer's Disease Genetics Consortium
Gail	Jarvik		PhD	University of Washington			Alzheimer's Disease Genetics Consortium
Suman	Jayadev		MD	University of Washington			Alzheimer's Disease Genetics Consortium
Lee-Way	Jin		MD, PhD	University of California Davis			Alzheimer's Disease Genetics Consortium
Kimberly	Johnson		MSW, PhD	Texas Tech University Health Science Center			Alzheimer's Disease Genetics Consortium

Supplemental Online Content: Nonauthor Collaborators
*First name, last name, and suffix (if applicable) are required and will appear in PubMed.

***First Name and Middle Initial(s)**	***Last Name**	***Suffix (eg, Jr, III)**	Academic Degrees	Institution	Location (city, state/province, country)	Role or Contribution, eg, chair, principal investigator	Group (if more than 1 Group listed in the byline) and/or Subgroup (eg, Steering Committee)
Leigh	Johnson		PhD	University of North Texas Health Science Center			Alzheimer's Disease Genetics Consortium
Gyungah	Jun		PhD	Boston University			Alzheimer's Disease Genetics Consortium
M. Ilyas	Kamboh		PhD	University of Pittsburgh			Alzheimer's Disease Genetics Consortium
Moon Il	Kang		PhD	Boston University			Alzheimer's Disease Genetics Consortium
Anna	Karydas		BA	University of California San Francisco			Alzheimer's Disease Genetics Consortium
Gauthreaux	Kathryn		PhD	University of Washington			Alzheimer's Disease Genetics Consortium
Mindy	Katz		MPH	Albert Einstein College of Medicine			Alzheimer's Disease Genetics Consortium
John	Kauwe		PhD	Brigham Young University			Alzheimer's Disease Genetics Consortium
Jeffery	Kaye		MD	Oregon Health & Science University			Alzheimer's Disease Genetics Consortium
C. Dirk	Keene		MD, PhD	University of Washington			Alzheimer's Disease Genetics Consortium
Benjamin	Keller		PhD	University of Washington			Alzheimer's Disease Genetics Consortium
Aisha	Khaleeq		MD	Baylor College of Medicine			Alzheimer's Disease Genetics Consortium
Ronald	Kim		MD	University of California Irvine			Alzheimer's Disease Genetics Consortium
Janice	Knebl		DO	University of North Texas Health Science Center			Alzheimer's Disease Genetics Consortium
Neil	Kowall		MD	Boston University			Alzheimer's Disease Genetics Consortium

Supplemental Online Content: Nonauthor Collaborators
*First name, last name, and suffix (if applicable) are required and will appear in PubMed.

***First Name and Middle Initial(s)**	***Last Name**	***Suffix (eg, Jr, III)**	Academic Degrees	Institution	Location (city, state/province, country)	Role or Contribution, eg, chair, principal investigator	Group (if more than 1 Group listed in the byline) and/or Subgroup (eg, Steering Committee)
Joel	Kramer		PsyD	University of California San Francisco			Alzheimer's Disease Genetics Consortium
Walter	Kukull		PhD	University of Washington			Alzheimer's Disease Genetics Consortium
Brian	Kunkle		PhD, MPH	University of Miami			Alzheimer's Disease Genetics Consortium
Amanda	Kuzma		MS	University of Pennsylvania Perelman School of Medicine			Alzheimer's Disease Genetics Consortium
Frank	LaFerla		PhD	University of California Irvine			Alzheimer's Disease Genetics Consortium
James	Lah		PhD	Emory University			Alzheimer's Disease Genetics Consortium
Eric	Larson		MD, MPH	University of Washington			Alzheimer's Disease Genetics Consortium
Melissa	Lerch		PhD	University of Washington			Alzheimer's Disease Genetics Consortium
Alan	Lerner		MD	Case Western Reserve University			Alzheimer's Disease Genetics Consortium
Yuk Ye	Leung		PhD	University of Pennsylvania Perelman School of Medicine			Alzheimer's Disease Genetics Consortium
James	Leverenz		MD	Cleveland Clinic			Alzheimer's Disease Genetics Consortium
Allan	Levey		MD, PhD	Emory University			Alzheimer's Disease Genetics Consortium
Donghe	Li		PhD	Boston University			Alzheimer's Disease Genetics Consortium
Andrew	Lieberman		MD, PhD	University of Michigan			Alzheimer's Disease Genetics Consortium
Richard	Lipton		MD	Albert Einstein College of Medicine			Alzheimer's Disease Genetics Consortium

Supplemental Online Content: Nonauthor Collaborators
*First name, last name, and suffix (if applicable) are required and will appear in PubMed.

*First Name and Middle Initial(s)	*Last Name	*Suffix (eg, Jr, III)	Academic Degrees	Institution	Location (city, state/province, country)	Role or Contribution, eg, chair, principal investigator	Group (if more than 1 Group listed in the byline) and/or Subgroup (eg, Steering Committee)
Oscar	Lopez		MD	University of Pittsburgh Alzheimer's Disease Research Center			Alzheimer's Disease Genetics Consortium
Kathryn	Lunetta		PhD	Boston University			Alzheimer's Disease Genetics Consortium
Constantine	Lyketsos		MD, MHS	Johns Hopkins University			Alzheimer's Disease Genetics Consortium
Douglas	Mains		DrPH	University of North Texas Health Science Center			Alzheimer's Disease Genetics Consortium
Jennifer	Manly		PhD	Columbia University			Alzheimer's Disease Genetics Consortium
Logue	Mark		PhD	Boston University			Alzheimer's Disease Genetics Consortium
David	Marquez		PhD	Rush University Medical Center			Alzheimer's Disease Genetics Consortium
Daniel	Marson		JD, PhD	University of Alabama at Birmingham			Alzheimer's Disease Genetics Consortium
Eden	Martin		PhD	University of Miami			Alzheimer's Disease Genetics Consortium
Eliezer	Masliah		MD	University of California San Diego			Alzheimer's Disease Genetics Consortium
Paul	Massman		PhD	Baylor College of Medicine			Alzheimer's Disease Genetics Consortium
Arjun	Masukar		MD, PhD	New York University			Alzheimer's Disease Genetics Consortium
Richard	Mayeux		MD	Columbia University			Alzheimer's Disease Genetics Consortium
Wayne	McCormick		MD, MPH	University of Washington			Alzheimer's Disease Genetics Consortium
Susan	McCurry		PhD	University of Washington			Alzheimer's Disease Genetics Consortium

*First name, last name, and suffix (if applicable) are required and will appear in PubMed.

*First Name and Middle Initial(s)	*Last Name	*Suffix (eg, Jr, III)	Academic Degrees	Institution	Location (city, state/province, country)	Role or Contribution, eg, chair, principal investigator	Group (if more than 1 Group listed in the byline) and/or Subgroup (eg, Steering Committee)
Stefan	McDonough		PhD	Pfizer Worldwide Research and Development			Alzheimer's Disease Genetics Consortium
Ann	McKee		MD	Boston University			Alzheimer's Disease Genetics Consortium
Marsel	Mesulam		MD	Northwestern University Feinberg School of Medicine			Alzheimer's Disease Genetics Consortium
Jesse	Mez		PhD	Boston University			Alzheimer's Disease Genetics Consortium
Bruce	Miller		MD	University of California San Francisco			Alzheimer's Disease Genetics Consortium
Carol	Miller		MD	University of Southern California			Alzheimer's Disease Genetics Consortium
Charles	Mock		PhD	University of Washington			Alzheimer's Disease Genetics Consortium
Abhay	Moghekar		MD	Johns Hopkins University			Alzheimer's Disease Genetics Consortium
Thomas	Montine		MD, PhD	University of Washington			Alzheimer's Disease Genetics Consortium
Edwin	Monuki			University of California Irvine			Alzheimer's Disease Genetics Consortium
Sean	Mooney		PhD	University of Washington			Alzheimer's Disease Genetics Consortium
John	Morris		MD	Washington University			Alzheimer's Disease Genetics Consortium
Shubhabrata	Mukherjee		PhD	University of Washington			Alzheimer's Disease Genetics Consortium
Amanda	Myers		PhD	University of Miami			Alzheimer's Disease Genetics Consortium
Adam	Naj		PhD	University of Pennsylvania Perelman School of Medicine			Alzheimer's Disease Genetics Consortium

Supplemental Online Content: Nonauthor Collaborators
*First name, last name, and suffix (if applicable) are required and will appear in PubMed.

*First Name and Middle Initial(s)	*Last Name	*Suffix (eg, Jr, III)	Academic Degrees	Institution	Location (city, state/province, country)	Role or Contribution, eg, chair, principal investigator	Group (if more than 1 Group listed in the byline) and/or Subgroup (eg, Steering Committee)
Trung	Nguyen		PhD	University of Texas Southwestern Medical Center			Alzheimer's Disease Genetics Consortium
Sid	O'Bryant		PhD	University of North Texas Health Science Center			Alzheimer's Disease Genetics Consortium
Kyle	Ormsby		PhD	University of Washington			Alzheimer's Disease Genetics Consortium
Marcia	Ory		PhD, MPH	Texas A&M University Health Science Center			Alzheimer's Disease Genetics Consortium
Raymond	Palmer		PhD	University of Texas Health Science Center - San Antonio			Alzheimer's Disease Genetics Consortium
Joseph	Parisi		MD	Mayo Clinic			Alzheimer's Disease Genetics Consortium
Henry	Paulson		MD, PhD	University of Michigan			Alzheimer's Disease Genetics Consortium
Valory	Pavlik		PhD	Baylor College of Medicine			Alzheimer's Disease Genetics Consortium
David	Paydarfar		MD	University of Texas at Austin/Dell Medical School			Alzheimer's Disease Genetics Consortium
Victoria	Perez			Texas Tech University Health Science Center			Alzheimer's Disease Genetics Consortium
Margaret	Pericak-Vance		PhD	University of Miami			Alzheimer's Disease Genetics Consortium
Ronald	Peterson		MD, PhD	Mayo Clinic			Alzheimer's Disease Genetics Consortium
Marsha	Polk			University of Texas Health Science Center - San Antonio			Alzheimer's Disease Genetics Consortium
Liming	Qu		MS	University of Pennsylvania Perelman School of Medicine			Alzheimer's Disease Genetics Consortium
Mary	Quiceno		MD	University of Texas Southwestern Medical Center			Alzheimer's Disease Genetics Consortium

Supplemental Online Content: Nonauthor Collaborators
*First name, last name, and suffix (if applicable) are required and will appear in PubMed.

***First Name and Middle Initial(s)**	***Last Name**	***Suffix (eg, Jr, III)**	Academic Degrees	Institution	Location (city, state/province, country)	Role or Contribution, eg, chair, principal investigator	Group (if more than 1 Group listed in the byline) and/or Subgroup (eg, Steering Committee)
Joseph	Quinn		MD	Oregon Health & Science University			Alzheimer's Disease Genetics Consortium
Ashok	Raj		MD	University of South Florida			Alzheimer's Disease Genetics Consortium
Farid	Rajabli		PhD	University of Miami			Alzheimer's Disease Genetics Consortium
Vijay	Ramanan		PhD	Mayo Clinic			Alzheimer's Disease Genetics Consortium
Eric	Reiman		MD	Translational Genomics Research Institute			Alzheimer's Disease Genetics Consortium
Joan	Reisch		PhD	University of Texas Southwestern Medical Center			Alzheimer's Disease Genetics Consortium
Christiane	Reitz		PhD	Columbia University			Alzheimer's Disease Genetics Consortium
John	Ringman		MD	University of Southern California			Alzheimer's Disease Genetics Consortium
Erik	Robertson		MD, PhD	University of Alabama at Birmingham			Alzheimer's Disease Genetics Consortium
Monica	Rodriguear		MA, MS	Baylor College of Medicine			Alzheimer's Disease Genetics Consortium
Ekaterina	Rogaeva		PhD	University of Toronto			Alzheimer's Disease Genetics Consortium
Howard	Rosen		MD	University of California San Francisco			Alzheimer's Disease Genetics Consortium
Roger	Rosenberg		MD	University of Texas Southwestern			Alzheimer's Disease Genetics Consortium
Donald	Royall		MD	South Texas Veterans Health Administration Geriatric Research Education & Clinical Cer			Alzheimer's Disease Genetics Consortium
Mary	Sano		PhD	Mount Sinai School of Medicine			Alzheimer's Disease Genetics Consortium

*First name, last name, and suffix (if applicable) are required and will appear in PubMed.

***First Name and Middle Initial(s)**	***Last Name**	***Suffix (eg, Jr, III)**	Academic Degrees	Institution	Location (city, state/province, country)	Role or Contribution, eg, chair, principal investigator	Group (if more than 1 Group listed in the byline) and/or Subgroup (eg, Steering Committee)
Andrew	Saykin		PsyD	Indiana University			Alzheimer's Disease Genetics Consortium
Gerard	Schellenberg		PhD	University of Pennsylvania Perelman School of Medicine			Alzheimer's Disease Genetics Consortium
Julie	Schneider		MD	Rush University Medical Center			Alzheimer's Disease Genetics Consortium
Lon	Schneider		MD	University of Southern California			Alzheimer's Disease Genetics Consortium
William	Seeley		MD	University of California San Francisco			Alzheimer's Disease Genetics Consortium
Richard	Sherva		PhD	Boston University			Alzheimer's Disease Genetics Consortium
Dean	Shibata		PhD	University of Washington			Alzheimer's Disease Genetics Consortium
Scott	Small		MD	Columbia University			Alzheimer's Disease Genetics Consortium
Amanda	Smith		MD	University of South Florida			Alzheimer's Disease Genetics Consortium
Janet	Smith		BS	University of Texas Southwestern Medical Center			Alzheimer's Disease Genetics Consortium
Yeunjoo	Song		PhD	Case Western Reserve University			Alzheimer's Disease Genetics Consortium
Salvatore	Spina		MD	Indiana University			Alzheimer's Disease Genetics Consortium
Peter	St George-Hyslop		MD, FRCP	University of Toronto			Alzheimer's Disease Genetics Consortium
Robert	Stern		PhD	Boston University			Alzheimer's Disease Genetics Consortium
Alan	Stevens		PhD	Texas A&M University Health Science Center			Alzheimer's Disease Genetics Consortium

Supplemental Online Content: Nonauthor Collaborators
*First name, last name, and suffix (if applicable) are required and will appear in PubMed.

***First Name and Middle Initial(s)**	***Last Name**	***Suffix (eg, Jr, III)**	Academic Degrees	Institution	Location (city, state/province, country)	Role or Contribution, eg, chair, principal investigator	Group (if more than 1 Group listed in the byline) and/or Subgroup (eg, Steering Committee)
Stephen	Strittmatter		MD, PhD	Yale University			Alzheimer's Disease Genetics Consortium
David	Sultzer			University of California Irvine			Alzheimer's Disease Genetics Consortium
Russell	Swerdlow		MD	University of Kansas Medical Center			Alzheimer's Disease Genetics Consortium
Jeffery	Tilson		PhD	University of North Carolina			Alzheimer's Disease Genetics Consortium
Giuseppe	Tosto		MD	Columbia University			Alzheimer's Disease Genetics Consortium
John	Trojanowski		MD, PhD	University of Pennsylvania Perelman School of Medicine			Alzheimer's Disease Genetics Consortium
Juan	Troncoso		MD	Johns Hopkins University			Alzheimer's Disease Genetics Consortium
Debby	Tsuang		MD	VA Puget Sound Health Care System/GRECC			Alzheimer's Disease Genetics Consortium
Otto	Valladares		MS	University of Pennsylvania Perelman School of Medicine			Alzheimer's Disease Genetics Consortium
Jeffery	Vance		MD, PhD	University of Miami			Alzheimer's Disease Genetics Consortium
Vivianna	Van Deerlin		MD, PhD	University of Pennsylvania Perelman School of Medicine			Alzheimer's Disease Genetics Consortium
Linda	Van Eldik		PhD	University of Kentucky			Alzheimer's Disease Genetics Consortium
Badri	Vardarajan		MS	Columbia University			Alzheimer's Disease Genetics Consortium
Robert	Vassar		PhD	Northwestern University Feinberg School of Medicine			Alzheimer's Disease Genetics Consortium
Harry	Vinters		MD	University of California Los Angeles			Alzheimer's Disease Genetics Consortium

Supplemental Online Content: Nonauthor Collaborators
*First name, last name, and suffix (if applicable) are required and will appear in PubMed.

*First Name and Middle Initial(s)	*Last Name	*Suffix (eg, Jr, III)	Academic Degrees	Institution	Location (city, state/province, country)	Role or Contribution, eg, chair, principal investigator	Group (if more than 1 Group listed in the byline) and/or Subgroup (eg, Steering Committee)
Jean Paul	Vonsattel		MD	Columbia University			Alzheimer's Disease Genetics Consortium
Li San	Wang		PhD	University of Pennsylvania Perelman School of Medicine			Alzheimer's Disease Genetics Consortium
Sandra	Weintraub		PhD	Northwestern University Feinberg School of Medicine			Alzheimer's Disease Genetics Consortium
Kathleen	Welsh-Bohmer		PhD	Duke University			Alzheimer's Disease Genetics Consortium
Nick	Wheeler		PhD	Case Western Reserve University			Alzheimer's Disease Genetics Consortium
Ellen	Wijsman		PhD	University of Washington			Alzheimer's Disease Genetics Consortium
Kirk	Wilhelmsen		MD, PhD	University of North Carolina Chapel Hill			Alzheimer's Disease Genetics Consortium
Scott	Williams		PhD	Case Western Reserve University			Alzheimer's Disease Genetics Consortium
Benjamin	Williams		MD	University of Texas Southwestern Medical Center			Alzheimer's Disease Genetics Consortium
Jennifer	Williamson		MS	Columbia University			Alzheimer's Disease Genetics Consortium
Henrick	Wilms		MD	Texas Tech University Health Science Center			Alzheimer's Disease Genetics Consortium
Thomas	Wingo		MD	Emory University			Alzheimer's Disease Genetics Consortium
Randall	Woltjer		MD, PhD	Oregon Health & Science University			Alzheimer's Disease Genetics Consortium
Martin	Woon		PhD	University of Texas at Austin/Dell Medical School			Alzheimer's Disease Genetics Consortium
Steven	Younkin		MD, PhD	Mayo Clinic			Alzheimer's Disease Genetics Consortium

Supplemental Online Content: Nonauthor Collaborators
*First name, last name, and suffix (if applicable) are required and will appear in PubMed.

***First Name and Middle Initial(s)**	***Last Name**	***Suffix (eg, Jr, III)**	Academic Degrees	Institution	Location (city, state/province, country)	Role or Contribution, eg, chair, principal investigator	Group (if more than 1 Group listed in the byline) and/or Subgroup (eg, Steering Committee)
Lei	Yu		PhD	Rush University Medical Center			Alzheimer's Disease Genetics Consortium
Yi	Zhao		MS	University of Pennsylvania Perelman School of Medicine			Alzheimer's Disease Genetics Consortium
Xiongwei	Zhou		PhD	Case Western Reserve University			Alzheimer's Disease Genetics Consortium
Congcong	Zhu			Boston University			Alzheimer's Disease Genetics Consortium
Olusegun	Adegoke		MSc				Alzheimer's Disease Neuroimaging Initiative
Paul	Aisen		MD				Alzheimer's Disease Neuroimaging Initiative
Liana G.	Apostolova		MD				Alzheimer's Disease Neuroimaging Initiative
Miriam	Ashford		PhD				Alzheimer's Disease Neuroimaging Initiative
Lisa	Barnes		PhD				Alzheimer's Disease Neuroimaging Initiative
Laurel	Beckett		PhD				Alzheimer's Disease Neuroimaging Initiative
Marie	Bernard		MD				Alzheimer's Disease Neuroimaging Initiative

Supplemental Online Content: Nonauthor Collaborators
*First name, last name, and suffix (if applicable) are required and will appear in PubMed.

*First Name and Middle Initial(s)	*Last Name	*Suffix (eg, Jr, III)	Academic Degrees	Institution	Location (city, state/province, country)	Role or Contribution, eg, chair, principal investigator	Group (if more than 1 Group listed in the byline) and/or Subgroup (eg, Steering Committee)
Haley	Bernhardt		BA, R. EEG T				Alzheimer's Disease Neuroimaging Initiative
Bret	Borowski		RTR				Alzheimer's Disease Neuroimaging Initiative
Yuliana	Cabrera		BS				Alzheimer's Disease Neuroimaging Initiative
Nigel J.	Cairns		PhD, FRCPath				Alzheimer's Disease Neuroimaging Initiative
Maria	Carrillo		PhD				Alzheimer's Disease Neuroimaging Initiative
Kewei	Chen		PhD				Alzheimer's Disease Neuroimaging Initiative
Mark	Choe		BS				Alzheimer's Disease Neuroimaging Initiative
Taylor	Clanton		MPH				Alzheimer's Disease Neuroimaging Initiative
Godfrey	Coker		MBA, MPH				Alzheimer's Disease Neuroimaging Initiative
Cat	Conti		BA				Alzheimer's Disease Neuroimaging Initiative

Supplemental Online Content: Nonauthor Collaborators
*First name, last name, and suffix (if applicable) are required and will appear in PubMed.

***First Name and Middle Initial(s)**	***Last Name**	***Suffix (eg, Jr, III)**	Academic Degrees	Institution	Location (city, state/province, country)	Role or Contribution, eg, chair, principal investigator	Group (if more than 1 Group listed in the byline) and/or Subgroup (eg, Steering Committee)
Karen	Crawford						Alzheimer's Disease Neuroimaging Initiative
Sandhitsu	Das		PhD				Alzheimer's Disease Neuroimaging Initiative
Charles	DeCarli		MD				Alzheimer's Disease Neuroimaging Initiative
Michael	Donohue		PhD				Alzheimer's Disease Neuroimaging Initiative
Kelley	Faber		MS, CCRC				Alzheimer's Disease Neuroimaging Initiative
Adam	Fleisher		MD				Alzheimer's Disease Neuroimaging Initiative
Derek	Flenneiken						Alzheimer's Disease Neuroimaging Initiative
Evan	Fletcher		PhD				Alzheimer's Disease Neuroimaging Initiative
Juliet	Fockler						Alzheimer's Disease Neuroimaging Initiative
Arvin	Forghanian-Arani		PhD				Alzheimer's Disease Neuroimaging Initiative

*First name, last name, and suffix (if applicable) are required and will appear in PubMed.

***First Name and Middle Initial(s)**	***Last Name**	***Suffix (eg, Jr, III)**	Academic Degrees	Institution	Location (city, state/province, country)	Role or Contribution, eg, chair, principal investigator	Group (if more than 1 Group listed in the byline) and/or Subgroup (eg, Steering Committee)
Tatiana M.	Foroud		PhD				Alzheimer's Disease Neuroimaging Initiative
Nick C.	Fox		MD				Alzheimer's Disease Neuroimaging Initiative
Erin	Franklin		MS				Alzheimer's Disease Neuroimaging Initiative
Devon	Gessert		BS				Alzheimer's Disease Neuroimaging Initiative
Hector	González						Alzheimer's Disease Neuroimaging Initiative
Robert C.	Green		MD, MPH				Alzheimer's Disease Neuroimaging Initiative
Jeffery	Gunter		PhD				Alzheimer's Disease Neuroimaging Initiative
Danielle	Harvey		PhD				Alzheimer's Disease Neuroimaging Initiative
Lindsey	Hergesheimer		BS				Alzheimer's Disease Neuroimaging Initiative
Carole	Ho						Alzheimer's Disease Neuroimaging Initiative

*First name, last name, and suffix (if applicable) are required and will appear in PubMed.

***First Name and Middle Initial(s)**	***Last Name**	***Suffix (eg, Jr, III)**	Academic Degrees	Institution	Location (city, state/province, country)	Role or Contribution, eg, chair, principal investigator	Group (if more than 1 Group listed in the byline) and/or Subgroup (eg, Steering Committee)
Erin	Householder		MS				Alzheimer's Disease Neuroimaging Initiative
John K.	Hsaio		MD				Alzheimer's Disease Neuroimaging Initiative
Clifford R.	Jack	Jr	MD				Alzheimer's Disease Neuroimaging Initiative
Jonathan	Jackson		PhD				Alzheimer's Disease Neuroimaging Initiative
William	Jagust		MD				Alzheimer's Disease Neuroimaging Initiative
Neda	Jahanshad		PhD				Alzheimer's Disease Neuroimaging Initiative
Gustavo	Jimenez		MBS				Alzheimer's Disease Neuroimaging Initiative
Chengshu	Jin		PhD				Alzheimer's Disease Neuroimaging Initiative
David	Jones		MD				Alzheimer's Disease Neuroimaging Initiative
Kejal	Kantarci		MD				Alzheimer's Disease Neuroimaging Initiative

*First name, last name, and suffix (if applicable) are required and will appear in PubMed.

***First Name and Middle Initial(s)**	***Last Name**	***Suffix (eg, Jr, III)**	Academic Degrees	Institution	Location (city, state/province, country)	Role or Contribution, eg, chair, principal investigator	Group (if more than 1 Group listed in the byline) and/or Subgroup (eg, Steering Committee)
Zaven	Khachaturian		PhD				Alzheimer's Disease Neuroimaging Initiative
Alexander	Knaack		MS				Alzheimer's Disease Neuroimaging Initiative
Robert A.	Koeppe		PhD				Alzheimer's Disease Neuroimaging Initiative
Adrienne	Kormos						Alzheimer's Disease Neuroimaging Initiative
Susan	Landau		PhD				Alzheimer's Disease Neuroimaging Initiative
Payam	Mahboubi		MPH				Alzheimer's Disease Neuroimaging Initiative
Ian	Malone		PhD				Alzheimer's Disease Neuroimaging Initiative
Eliezer	Masliah		MD				Alzheimer's Disease Neuroimaging Initiative
Donna	Masterman		<D				Alzheimer's Disease Neuroimaging Initiative
Chet	Mathis		MD				Alzheimer's Disease Neuroimaging Initiative

Supplemental Online Content: Nonauthor Collaborators
*First name, last name, and suffix (if applicable) are required and will appear in PubMed.

***First Name and Middle Initial(s)**	***Last Name**	***Suffix (eg, Jr, III)**	Academic Degrees	Institution	Location (city, state/province, country)	Role or Contribution, eg, chair, principal investigator	Group (if more than 1 Group listed in the byline) and/or Subgroup (eg, Steering Committee)
Garrett	Miller		MS				Alzheimer's Disease Neuroimaging Initiative
Tom	Montine		MD, PhD				Alzheimer's Disease Neuroimaging Initiative
Shelley	Moore		BA				Alzheimer's Disease Neuroimaging Initiative
John C.	Morris		MD				Alzheimer's Disease Neuroimaging Initiative
Scott	Neu		PhD				Alzheimer's Disease Neuroimaging Initiative
John	Neuhaus		PhD				Alzheimer's Disease Neuroimaging Initiative
Kwangsik	Nho		PhD				Alzheimer's Disease Neuroimaging Initiative
Talia M.	Nir		PhD				Alzheimer's Disease Neuroimaging Initiative
Rachel	Nosheny		PhD				Alzheimer's Disease Neuroimaging Initiative
Kelly	Nudelman		PhD				Alzheimer's Disease Neuroimaging Initiative

*First name, last name, and suffix (if applicable) are required and will appear in PubMed.

***First Name and Middle Initial(s)**	***Last Name**	***Suffix (eg, Jr, III)**	Academic Degrees	Institution	Location (city, state/province, country)	Role or Contribution, eg, chair, principal investigator	Group (if more than 1 Group listed in the byline) and/or Subgroup (eg, Steering Committee)
Ozioma	Okonkwo		PhD				Alzheimer's Disease Neuroimaging Initiative
Richard J.	Perrin		MD, PhD				Alzheimer's Disease Neuroimaging Initiative
Ronald	Peterson		MD, PhD				Alzheimer's Disease Neuroimaging Initiative
Jeremy	Pizzola		BA				Alzheimer's Disease Neuroimaging Initiative
William	Potter		MD				Alzheimer's Disease Neuroimaging Initiative
Michael	Rafii		MD, PhD				Alzheimer's Disease Neuroimaging Initiative
Rema	Raman		PhD				Alzheimer's Disease Neuroimaging Initiative
Robert	Reid		PhD				Alzheimer's Disease Neuroimaging Initiative
Eric R.	Reiman		MD				Alzheimer's Disease Neuroimaging Initiative
Shannon L.	Risacher		PhD				Alzheimer's Disease Neuroimaging Initiative

*First name, last name, and suffix (if applicable) are required and will appear in PubMed.

***First Name and Middle Initial(s)**	***Last Name**	***Suffix (eg, Jr, III)**	Academic Degrees	Institution	Location (city, state/province, country)	Role or Contribution, eg, chair, principal investigator	Group (if more than 1 Group listed in the byline) and/or Subgroup (eg, Steering Committee)
Stephanie	Rossi Chen		BA				Alzheimer's Disease Neuroimaging Initiative
Laurie	Ryan		PhD				Alzheimer's Disease Neuroimaging Initiative
Jennifer	Salazar		MBS				Alzheimer's Disease Neuroimaging Initiative
Andrew J.	Saykin		PsyD				Alzheimer's Disease Neuroimaging Initiative
Christopher	Schwarz		PhD				Alzheimer's Disease Neuroimaging Initiative
Matthew	Senjem		MS				Alzheimer's Disease Neuroimaging Initiative
Elizabeth	Shaffer		BS				Alzheimer's Disease Neuroimaging Initiative
Leslie M.	Shaw		PhD				Alzheimer's Disease Neuroimaging Initiative
Li	Shen		PhD				Alzheimer's Disease Neuroimaging Initiative
Nina	Silverberg		PhD				Alzheimer's Disease Neuroimaging Initiative

Supplemental Online Content: Nonauthor Collaborators
*First name, last name, and suffix (if applicable) are required and will appear in PubMed.

***First Name and Middle Initial(s)**	***Last Name**	***Suffix (eg, Jr, III)**	Academic Degrees	Institution	Location (city, state/province, country)	Role or Contribution, eg, chair, principal investigator	Group (if more than 1 Group listed in the byline) and/or Subgroup (eg, Steering Committee)
Stephanie	Smith		BS				Alzheimer's Disease Neuroimaging Initiative
Lisa	Taylor-Reinwald		BA, HTL				Alzheimer's Disease Neuroimaging Initiative
Leon	Thal		MD				Alzheimer's Disease Neuroimaging Initiative
Sophia I.	Thomopoulos		BS				Alzheimer's Disease Neuroimaging Initiative
Paul	Thompson		PhD				Alzheimer's Disease Neuroimaging Initiative
Arthur W.	Toga		PhD				Alzheimer's Disease Neuroimaging Initiative
Duygu	Tosun-Turgut		PhD				Alzheimer's Disease Neuroimaging Initiative
John Q.	Trojanowski		MD, PhD				Alzheimer's Disease Neuroimaging Initiative
Diana	Truran Sacrey						Alzheimer's Disease Neuroimaging Initiative
Dallas	Veitch		PhD				Alzheimer's Disease Neuroimaging Initiative

Supplemental Online Content: Nonauthor Collaborators
*First name, last name, and suffix (if applicable) are required and will appear in PubMed.

***First Name and Middle Initial(s)**	***Last Name**	***Suffix (eg, Jr, III)**	Academic Degrees	Institution	Location (city, state/province, country)	Role or Contribution, eg, chair, principal investigator	Group (if more than 1 Group listed in the byline) and/or Subgroup (eg, Steering Committee)
Prashanthi	Vemuri		PhD				Alzheimer's Disease Neuroimaging Initiative
Sarah	Walter		MSc				Alzheimer's Disease Neuroimaging Initiative
Chad	Ward						Alzheimer's Disease Neuroimaging Initiative
Michael W.	Weiner		MD				Alzheimer's Disease Neuroimaging Initiative
Kristi	Wilmes		MS, CCRP				Alzheimer's Disease Neuroimaging Initiative
Paul A.	Yushkevich		PhD				Alzheimer's Disease Neuroimaging Initiative
Caileigh	Zimmerman		MS				Alzheimer's Disease Neuroimaging Initiative
