# Supplementary material for: Associations of Sex, Race, and Apolipoprotein E Alleles With Multiple Domains of Cognition Among Older Adults
Source: JAMA Neurol. 2023 Jul 17;80(9):929–39. doi: 10.1001/jamaneurol.2023.2169 (PMC10352930; doi:10.1001/jamaneurol.2023.2169)
Supplement: Supplement 4. — Data sharing statement [file jamaneurol-e232169-s004.pdf]

Data Sharing Statement

Data
Data available: Yes
Data types: Other (please specify)
Additional Information: Data is publicly available to qualified investigators.
How to access data: Data from ADNI is shared through the LONI Image and Data Archive (https://ida.loni.usc.edu/). Data from ROS/MAP/MARS can be requested at www.radc.rush.edu. Data from NACC can be accessed at https://naccdata.org/. Data from ACT can be accessed through the Data Query Tool (http://act.kpwashingtonresearch.org/dqt/).
When available: With publication

Supporting Documents
Document types: None

Additional Information
Who can access the data: Data will be made available to qualified investigators with IRB approval.
Types of analyses: Data will be made available to any qualified investigator.
Mechanisms of data availability: Data will be made available following data access approval through our data portal (https://www.vmacdata.org/vmap/data-requests).
